# THE CLARK TIBBITTS AWARD AND HIRAM J. FRIEDSAM AWARD LECTURES AND PRESENTATIONS

**DOI:** 10.1093/geroni/igae098.1245

**Published:** 2024-12-31

**Authors:** Joann Montepare

**Affiliations:** Chair

## Abstract

The Clark Tibbitts Award Lecture will feature an address by the 2024 award recipient Joann M. Motepare, PhD, FGSA, FAGHE, of Lasell University. The Hiram J. Friedsam Award Lecture will feature an address by the 2024 award recipient Dana Burr Bradley, PhD, FGSA, FAGHE, of the University of Maryland Baltimore County. AGHE’s Clark Tibbitts Award was established in 1980 and named for an architect of the field of gerontological education. The award is given each year to an individual or organization that has made an outstanding contribution to the advancement of gerontology and geriatrics education. Hiram J. Friedsam was the professor, co-founder, and director of the Center for Studies in Aging and dean of the School of Community Service at the University of Northern Texas. Dr. Friedsam was an outstanding teacher, researcher, colleague, and mentor to students, faculty, and administrators, as well as a past president of AGHE. The purpose of this award is to recognize those who emulate Dr. Friedsam’s excellence in mentorship.

